# Vitamin C Deficiency in Patients With Acute Myeloid Leukemia

**DOI:** 10.3389/fonc.2022.890344

**Published:** 2022-06-27

**Authors:** Tiziana Ottone, Isabella Faraoni, Giorgio Fucci, Mariadomenica Divona, Serena Travaglini, Eleonora De Bellis, Francesco Marchesi, Daniela Francesca Angelini, Raffaele Palmieri, Carmelo Gurnari, Manuela Giansanti, Anna Maria Nardozza, Federica Montesano, Emiliano Fabiani, Elisa Linnea Lindfors Rossi, Raffaella Cerretti, Laura Cicconi, Marco De Bardi, Maria Luisa Catanoso, Luca Battistini, Renato Massoud, Adriano Venditti, Maria Teresa Voso

**Affiliations:** ^1^ Department of Biomedicine and Prevention, University of Rome Tor Vergata, Rome, Italy; ^2^ Neuro-Oncohematology Unit, Istituto di Ricovero e Cura a Carattere Scientifico (IRCCS) Fondazione Santa Lucia, Rome, Italy; ^3^ Department of Systems Medicine, University of Rome Tor Vergata, Rome, Italy; ^4^ Department of Experimental Medicine and Surgery, Faculty of Medicine and Surgery, University Tor Vergata, Rome, Italy; ^5^ UniCamillus‐Saint Camillus International University of Health Sciences, Rome, Italy; ^6^ Department of Biomedicine and Prevention, PhD in Immunology, Molecular Medicine and Applied Biotechnology, University of Rome Tor Vergata, Rome, Italy; ^7^ Struttura Complessa (SC) Ematologia, Azienda Sanitaria Universitaria Giuliano Isontina Trieste, Trieste, Italy; ^8^ Hematology and Stem Cell Transplant Unit, Istituto di Ricovero e Cura a Carattere Scientifico (IRCCS) Regina Elena National Cancer Institute, Rome, Italy; ^9^ Neuroimmunology Unit, Istituto di Ricovero e Cura a Carattere Scientifico (IRCCS) Santa Lucia Foundation, Rome, Italy; ^10^ Department of Translational Hematology and Oncology Research, Taussig Cancer Institute, Cleveland Clinic, Cleveland, OH, United States; ^11^ Ospedale Santo Spirito, Azienda Sanitaria Locale (ASL) Roma 1, Reparto di Ematologia, Rome, Italy; ^12^ Department Hematology/Oncology and Cell and Gene Therapy, Istituto di Ricovero e Cura a Carattere Scientifico (IRCCS) Ospedale Pediatrico Bambino Gesú, Rome, Italy

**Keywords:** acute myeloid leukemia, vitamin C, vitamin C transporters, ascorbate uptake, NGS - next generation sequencing

## Abstract

Vitamin C has been shown to play a significant role in suppressing progression of leukemia through epigenetic mechanisms. We aimed to study the role of vitamin C in acute myeloid leukemia (AML) biology and clinical course. To this purpose, the plasma levels of vitamin C at diagnosis in 62 patients with AML (including 5 cases with acute promyelocytic leukemia, APL),7 with myelodysplastic syndrome (MDS), and in 15 healthy donors (HDs) were studied. As controls, vitamins A and E levels were analysed. Expression of the main vitamin C transporters and of the *TET2* enzyme were investigated by a specific RQ-PCR while cytoplasmic vitamin C concentration and its uptake were studied in mononuclear cells (MNCs), lymphocytes and blast cells purified from AML samples, and MNCs isolated from HDs. There were no significant differences in vitamin A and E serum levels between patients and HDs. Conversely, vitamin C concentration was significantly lower in AML as compared to HDs (p<0.0001), inversely correlated with peripheral blast‐counts (p=0.029), significantly increased at the time of complete remission (CR) (p=0.04) and further decreased in resistant disease (p=0.002). Expression of the main vitamin C transporters *SLC23A2*, *SLC2A1* and *SLC2A3* was also significantly reduced in AML compared to HDs. In this line, cytoplasmic vitamin C levels were also significantly lower in AML-MNCs versus HDs, and in sorted blasts compared to normal lymphocytes in individual patients. No association was found between vitamin C plasma levels and the mutation profile of AML patients, as well as when considering cytogenetics or 2017 ELN risk stratification groups. Finally, vitamin C levels did not play a predictive role for overall or relapse-free survival. In conclusion, our study shows that vitamin C levels are significantly decreased in patients with AML at the time of initial diagnosis, further decrease during disease progression and return to normal upon achievement of CR. Correspondingly, low intracellular levels may mirror increased vitamin C metabolic consumption in proliferating AML cells.

## Introduction

Vitamins are organic molecules required for the proper functioning of human metabolism. In particular, vitamins A, E and C display antioxidant properties against free radicals, which are known to cause cell damage and contribute to many diseases (1–3). Several studies have recently investigated the level of different vitamins in hematological malignancies, in particular in the context of myeloid neoplasms (4, 5). Acute myeloid leukemia (AML) is a heterogeneous disease with variable response to therapy, due to the intrinsic genetic complexity already present at diagnosis and/or evolving during the disease course because of therapeutic pressure and clonal evolution dynamics (6). Advances in DNA sequencing technologies have shed light on the genomic and epigenomic landscapes in AML (7, 8), demonstrating that proteins involved in epigenetic processes along with genetic mutations may contribute to disease onset (9). Emerging evidence identified vitamin C as a critical regulator of cellular epigenetic processes, in particular for its key role in the activation of ten-eleven translocation (TET) dioxygenases to enhance demethylation of DNA and histones (10).

Besides its role in epigenetics, vitamin C is a pivotal regulator of many physiological processes in humans (11), particularly during cellular immune responses (12). Specifically, in this latter scenario, vitamin C at physiological concentrations (<0.1 mM) represents an antioxidant molecule able to scavenge reactive oxygen species in order to prevent DNA damage and other effects, which ultimately may lead to cancer transformation. Unlike most mammals, humans are unable to generate endogenous vitamin C from oxidated glucose due to the lack of gulonolactone (L-) oxidase (GULO), and are thereby dependent on dietary intake (13). Vitamin C exists in two main forms, ascorbate (reduced active form) and dehydro-ascorbic acid (DHA, oxidized form), the latter with effective antioxidant features, acting as a free radical scavenger and an essential cofactor in numerous enzymatic reactions (14). In physiologic condition, vitamin C is present in plasma mainly as ascorbate and is actively absorbed in the gastrointestinal tract by the sodium-dependent vitamin C transporters SVCT1 and SVCT2, encoded by SLC23A1 and SLC23A2 genes, respectively (13, 15). However, vitamin C may enter into the cells also as DHA via the ubiquitous glucose transporters GLUT1 and GLUT3, encoded by the SLC2A1 and SLC2A3 genes, respectively (16).

The putative role of vitamin C in the development of cancer has been discussed for decades (17). Its deficiency is common in patients with advanced neoplastic disorders, in particular after chemotherapy and hematopoietic stem cells (HSCs) transplantation, and is associated with shorter survival (18, 19). An increased HSCs compartment characterized by enhanced self-renewal capabilities has been found in animal models with vitamin C deficiency and has been correlated with a predisposition to leukemic transformation (20).

At pharmacological dose vitamin C functions as a pro-oxidant. Vitamin C has been suggested as a potential anticancer agent, and several preclinical data have recently confirmed its selective cytotoxicity in different human malignancies, both in vitro and in vivo (21–23). Recently, pharmacological studies have demonstrated that vitamin C may represents a potent anti-cancer agent when administered intravenously and in high doses (24). In this line, Cimmino and colleagues (25), showed that treatment with vitamin C mimics TET2 restoration in Tet2-deficient mouse HSPCs, suppressing human leukemic colony formation, and leukemia progression in primary human leukemia patient-derived xenografts (PDXs). In addition to TET2, many enzymes are involved in DNA demethylation and have been shown to require vitamin C to maintain a proper activity (10). This concept has an important implication for AML patients because of the role of demethylase enzymes in the development and progression of AML (26).

To our knowledge, few studies have evaluated the levels of vitamin C in AML patients at disease onset. Herein, we investigated the levels of vitamin C in plasma and within primary blasts in a large cohort of AML patients at baseline and during the disease course both at achievement of complete remission (CR) and at disease progression.

## Materials and Methods

### Patients

Our study cohort included 57 consecutive cases of AML (median age: 68 years, range 29–83, 23 female and 34 male), 5 patients with acute promyelocytic leukemia (APL) (median age: 57 years, range 44–73, 3 female and 2 male) and 7 with myelodysplastic syndrome (MDS) (median age: 82 years, range 74–88, 1 female and 6 male) diagnosed and treated at the Hematology Unit of Policlinico Tor Vergata, Rome, Italy, during the years 2017-2020. AML patients aged <75 years received intensive chemotherapy according to ELN-2017 (20) and Gruppo Italiano Malattie EMatologiche dell’Adulto (GIMEMA) protocol (27), while patients aged >75 years received hypomethylating treatment or supportive care according to their performance status. Based on established risk criteria (28)), APL patients were treated with ATRA plus standard chemotherapy (n=1) or with the all trans-retinoic acid (ATRA) and arsenic trioxide (ATO) combination (n=4) as shown previously (29). Based on IPSS-R at diagnosis (30), MDS patients were classified as lower-risk (LR, ≤ 3.5; n=2) and higher-risk (HR, > 3.5;n=5) and were treated according to the European Society for Medical Oncology (ESMO) guidelines with either supportive measures or disease-modifying agents, respectively (31, 32). Fifteen healthy donors (HDs) were also included as a control group (median age: 48 years, range 33–66). Clinical and biological features of our patient cohort are shown in **Table 1**. According to the declaration of Helsinki, all patients and controls gave informed consent to the study, which was approved by our Institutional Review Board.

**Table 1:** Clinic-biological features of patients.

	AML	APL
Features	N (%)	
Median age (range)	68 (29-83)	57 (44-73)
Sex (M/F)	34/23	2/3.
BM blasts % (median, range)	51 (20-95)	84 (73-90)
PB blasts % (median, range)	28 (0-89)	48 (25-70)
WBC n/mcL (median, range)	8930 (10-83200)	2260 (1090-33300)
Cytogenetics (^)		
Normal	23 (41)	
Del/mon	13 (23.2)	
Recurrent	6 (10.7)	5 (100)
CK	8 (14.4)	
na (^)		
Other	6 (10.7)	
*FLT3* status		
mut	15 (26.3)	
wt	42 (73.7)	
*NPM1* status		
mut	15 (26.3)	
wt	42 (73.7)	
ELN 2017 prognostic categories (^^)		
Favourable	16 (31.4)	5 (100)
Intermediate	18 (35.3)	/
Adverse	17 (33.3)	/
Response to first treatment		
CR	24 (42.1)	5 (100)
Refractory	29 (50.9)	/
Not evaluable	4 (7)	/
Clinical outcome		
Relapsed	13 (22.8)	0 (100)
Alive	26 (49.1)	5 (100)
Dead	29 (50.9)	/
Median follow-up (days, range)	241 (11-965)	630 (390-1230)

WBC, white blood count; mut, mutated; wt, wild type; del/mon, deletion, monosomy; CK, complex karyotype; ELN, European Leukemia Net; CR, complete remission. (^) data not available in one patient (^^) data not available in 6 patients.

### Diagnostic Work-Up

Routine morphologic, immunophenotypic and genetic analyses were carried out in all patients at presentation using standard techniques, as detailed elsewhere (27, 33). Conventional karyotyping was performed on bone marrow (BM) diagnostic aspirates after short-term culture and analyzed after G-banding. The description of the karyotypes was done according to the International System for Human Cytogenetic Nomenclature (34). As for molecular analysis, total RNA was extracted from Lympholyte-H (Cedarlane) isolated BM and peripheral (PB) mononuclear cells (MNCs) collected at diagnosis and in selected cases during follow-up using standard procedures, and reverse-transcribed with random hexamers as primers (35). Molecular diagnostic analyses for recurrent gene translocations were performed in all AML patients at the time of initial diagnosis (36, 37). DNA was extracted using a column based Qiagen kit protocol (Qiagen, Hilden, Germany). Mutations of NPM1 and FLT3 genes were investigated at diagnosis on DNA extracted from BM-MNCs, using protocols reported elsewhere (38, 39). Patients’ genetic and cytogenetic features were used to stratify our population according to the 2017 ELN guideline version.

### High Speed Cell Sorting of Leukemic Blasts

Based on availability of fresh primary AML blasts, we selected for further analysis 8 AML samples at diagnosis. MNCs samples were separated using Ficoll gradient. The cells were then labeled with the following anti-human antibodies: CD45 APC Vio 770 (Miltenyi Biotec); CD3 and CD19 Brilliant Violet 780, CD34 APC and CD33 PE-CF594 (Becton Dickinson); CD14 PE-Cy5.5 (Beckman Coulter). In order to exclude dead cells, we used the LIVE/DEAD Fixable Aqua Dead Cell Stain Kit (Invitrogen). The different populations were sorted by high-speed cell sorting, in the 6-way sorting MoFlo Astrios (Beckman Coulter) using a sequential gating strategy described in **Supplementary Figure 1**. After doublets and dead cells exclusion, lymphocyte and monocyte population were used as controls and leukemia cells were selected for CD34 or CD33 expression.

### Analysis of Vitamin A, E and C Levels

Evaluation of the vitamin A, E and C was performed by isocratic high performance liquid chromatographic method with UV detection from serum and plasma. Vitamins purification was carried out using commercially available kits from Chromsystems Instruments & Chemicals GmbH (Grafälfing, Germany).

### Expression Analysis of Vitamin C Transporters SLC2A1, SCL23A2, SLC2A3 and TET2 Enzyme

We designed specific quantitative TaqMan realtime PCR assays (RQ-PCR) for SLC2A1, SCL23A2, SLC2A3 and TET2 transcript quantification. For the generation of plasmid standard curves, RT-PCR products of each transcript were cloned into the plasmid vector pCR II-TOPO (Invitrogen, Groningen, The Netherlands). After a blue/white selection, plasmid DNAs were purified by Nucleospin Plasmid DNA purification kit (Macherey-Nagel) from recombinant colonies, and sequenced using the bigdye® terminator v3·1 cycle sequencing kit (Thermo Fisher Scientific). Sequencing analysis was performed on an ABI 3130 automated sequencer (Thermo Fisher Scientific), and data was analyzed using the seqscape software version 2·5. Plasmid DNA concentration was determined by absorbance measurement and 6 serial plasmid dilutions (107, 106, 105, 104, 103, 102 copies) were prepared for RQ-PCR assays in order to generate a standard curve. Primer and probe sequences used in this study (designed using the Software Primer Express, Thermo Fisher Scientific, Foster City CA, USA) are shown in **Table 2**. RQ-PCR reactions and fluorescence measurements were carried out on the ABI PRISM 7700 Sequence Detection System (Thermo Fisher Scientific). Briefly, the reaction mixture of 25 µl contained 1×Master Mix (Thermo Fisher Scientific, Foster City, CA), 300 nM of each primer, 200 nM of Taqman probe and 5 µl of cDNA. Amplification conditions were: 2 min at 50°C, 10 min at 95°C followed by 60 cycles at 95°C for 15 s and at 60°C for 1 min. A threshold value of 0.2 was used and baseline was set to 3-15. The level of each transcript was normalized on the number of Abelson (ABL1) gene and expressed as copies number every 104 copies of ABL1. Following serial dilution experiments with water, the quantitative assays showed maximum reproducible sensitivity at 10-4 for SLC2A1, SLC23A2 and TET2, and 10-5 for SLC2A3 transcripts. The coefficient of the standard curves for the 4 assays ranged from 0.998 to 0.999 and the slope ranged from 3.296 to 3.359.

**Table 2:** Primer and probe sequences used for *TET2*, *SLC2A1*, *SCL23A2* and *SLC2A3* QRT-PCR assay.

Gene	Primer Forward	Primer Reverse	Probe	Tm °C	Application
***TET2***	TET2-F 5’-GATGGCTGCCCTTTAGGATTTGTTAGAA-3’	TET2-R 5’-CTGCTCTTCCTGGATCATGTCCTATT-3’	/	59	Plasmid construction
	TET2-FQ 5’-CGAGGCTGGCAAACATTCA-3’	TET2-RQ 5’-GGAGCAAAGGCAAGTAAACAATC-3’	TET2 5’-FAM-CAGCACACCCTCT-BHQ1-3’	60	QRT-PCR
***SLC2A1***	SLC2A1-F 5’-TCCTTCTCTGTGGGCCTTTTCGTTA-3’	SLC2A1-R 5’-ACACTTCACCCACATACATGGGCACGAA-3’	/	60	Plasmid construction
	SLC2A1-FQ 5’-CCGTGCTCATGGGCTTCT-3’	SLC2A1-RQ 5’-GCCCAGGATCAGCATCTCAA-3’	SLC2A1 5’-FAM-AAACTGGGCAAGTCC-BHQ1-3’	60	QRT-PCR
***SLC23A2***	SLC23A2-F 5’-ACGGCTGTGTAAACTACTCGTTTCTCTTA-3’	SLC23A2-R 5’-TTTTCTGCAATGCCGTTTTCCGTAGTGTA-3’	/	59	Plasmid construction
	SLC23A2-FQ 5’-ATGGAGGCTGGAAGTTCAACA-3’	SLC23A2-RQ 5’-GAGTGAAGAAAGCTGGGTGCTT-3’	SLC23A2 5’-FAM-AAGGCAAATACGAAGACG-BHQ1-3’	60	QRT-PCR
***SLC2A3***	SLC2A3-F 5’-TTTGGCAGGCGCAATTCAATGCTGATT-3’	SLC2A3-R 5’-CAACCGCTGGAGGATCTGCTTAGCAT-3’	/	59	Plasmid construction
	SLC2A3-FQ 5’-TGCGGACTCTGCACAGGTT-3’	SLC2A3-RQ 5’-AGGGCAGTAGGCGAGATCTCT-3’	SLC2A3 5’-FAM-TGTGCCCATGTACATTG-BHQ1-3’	60	QRT-PCR

REFSEQ mRNA: TET2 NM_001127208.3, SLC2A1 NM_006516.4, SLC23A2 NM_005116.6, SLC2A3 NM_006931.3.

### Targeted NGS Analyses

To assess the mutational profile of AML patients included in the study, we evaluated 30 genes, known to be frequently mutated in myeloid malignancies, using NGS and the Myeloid Solution by SOPHiA GENETICS (Saint-Sulpice, Switzerland). The resulting captured libraries were further processed on a MiniSeq sequencing platform (Illumina, San Diego, CA, USA). FASTQ sequencing files were then uploaded on the SOPHiA DDM platform for analysis by the specific technology. Only alterations identified as highly or potentially pathogenic were considered for analysis. The sensitivity of this NGS assay was ≥ 1%. The FASTQ files were further processed using the Sequence Pilot software version 4.1.1 (JSI Medical Systems) for alignment and variant calling. The functional significance of the somatic mutations was checked on the public Catalogue Of Somatic Mutations In Cancer (COSMIC) v69 database. Functional interpretation was performed using SIFT 1.03, PolyPhen 2.0 and MutationTaster 1.0 algorithms. Single‐nucleotide polymorphisms (SNPs), annotated according to the National Center for Biotechnology Information Single Nucleotide Polymorphism Database (NCBI dbSNP) were deleted from the analysis. The detection limit for variants was 5% variant allele frequency (VAF).

### Cell Culture and Ascorbate Treatment

Primary PB-MNCs of 5 AML patients with blast proportion ≥ 60% and PB-MNCs from 2 healthy donors, were selected for culture studies. Briefly, 106 primary AML and HDs cells/ml were seeded into a culture flask and cultured in RPMI-1640 medium (Sigma-Aldrich, St. Louis, MO, USA) supplemented with 2 glutamine (EuroClone, Pero, Milan, Italy), 1% penicillin/streptomycin (Euroclone), 20% FBS (Signa-Aldrich), 10 ng/ml each of IL-3, SCF and FLT3LG (Peprotech) at 37°C in a humidified CO2 incubator.

Ascorbate (L-ascorbate, Sigma-Aldrich) was diluted in RPMI-1640 medium at 250 mM concentration. Drug aliquots were stored at −80°C and for each experiment a new aliquot was thawed and used at a final concentration of 1mM. Cultured cells and supernatant were collected at indicated times and analysed for vitamin C content. Ascorbate 1mM corresponds to 198.1 mg/L of ascorbate or 176.12 mg/L of vitamin C in its oxidized form.

### Vitamin C Uptake in Primary AML Blasts and in Healthy Individuals

To study intracellular vitamin C content, primary PB-MNCs from AML and healthy individuals pellets (range 0.5-5 x106 cells) were lysed on ice using 100 µl of ice-cold internal solution (Chromsystems Instruments) for 20 min before centrifugation at 13,000 rpm for 10 min at 4°C. The supernatant was collected and stored at -80°C until HPLC analysis. The amount of total vitamin C in the 100 µl of extraction buffer (internal standard) was divided by the number of cells, and expressed in femtomol/cell.

### Statistical Analysis

Statistical analysis was performed by means of GraphPad Prism software version 5.0 (LaJolla, CA). Non parametric tests (Mann Whitney and Kruskal-Wallis) were used to evaluate the association between vitamin A, E and C levels and patient characteristics (age, sex, karyotype, FLT3 and NPM1 mutational status, and 2017-ELN risk groups). Correlations between vitamin A, E and C levels, and white blood cells (WBC) or BM/PB blast counts was carried out using the Spearman method and the relative Rho coefficient. Survival curves were estimated according to the Kaplan-Meier method and were tested for significance by the log-rank test. All tests were 2-sided, accepting p<0.05 to indicate statistically significant differences, and confidence intervals were calculated at 95% level. Survival curves were built by using the software package SPSS version 17.0 (Chicago, IL).

## Results

### Circulating Levels of Vitamin A, E and C in Patients With Myeloid Neoplasms and Healthy Individuals

**Figure 1:**
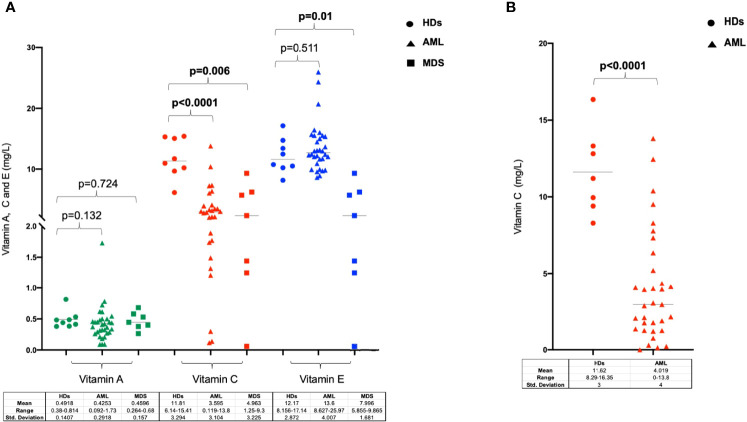
Levels of Vitamin A, C and E in patients with myeloid neoplasms and in healthy donors. **(A)** Vitamin A, C and E concentration analyzed in serum or plasma in healthy individuals, AML and MDS patients at diseases diagnosis. **(B)** Analysis of Vitamin C plasma levels in a validation cohort of healthy donors and AML patients at disease onset. HDs, healthy donors.

We initially analysed the PB levels of vitamins A, C and E in a cohort of 29 AML (including 5 APL cases) and 7 MDS patients at diagnosis and in 8 HDs. As shown in **Figure 1A**, there were no significant differences in vitamin A and E levels between patient with myeloid neoplasms and HDs, except for lower level of vitamin E observed in MDS as compared with HDs (p=0.01). Conversely, vitamin C concentration was significantly lower in patients with AML and MDS as compared to controls (AML p<0.0001 and MDS p=0.006). Based on these preliminary results, we extended the analysis of vitamin C plasma level to a validation cohort including additional 7 HDs and 33 AML samples at diagnosis confirming the lower level of circulating vitamin C in AML patients at onset (p<0.0001, **Figure 1B**).

### Correlations Between Plasma Vitamin C Levels, AML Biological Features, and Treatment Response

Correlations between vitamin C level and clinical-biological characteristics were studied in the total cohort of 62 AML samples at diagnosis (**Table 1**). Conventional karyotype or fluorescence in situ hybridization (FISH) were evaluable in 61/62 AML (98%) and 7 MDS (100%) cases. NPM1 and FLT3-ITD mutations were present in 15 of 57 AML cases (26.3% for both).

**Figure 2:**
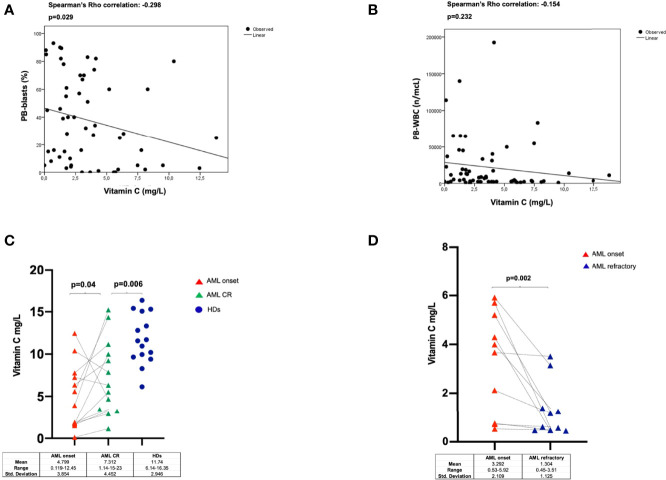
Correlations between Vitamin C levels and PB-blast proportion **(A)**, or WBC counts **(B)** in 62 AML patients (including 5 APL cases). Vitamin C plasma levels evaluated in AML patients at diagnosis and at the time of complete remission (paired samples) and in HDs **(C)** and at disease progression (refractory) (paired diagnostic and relapse samples) **(D)**. HDs, healthy donors; CR, complete remission.

No significant correlations were found between vitamin C plasma concentrations, sex (p=0.851) and age in AML patients (p=0.104, Spearman’s Rho correlation: -0.218) and HDs (sex p=0.77, Spearman’s Rho correlation: -0.009; age p=0.84, Spearman’s Rho correlation: -0.05). There was an inverse correlation between circulating vitamin C levels and PB blast proportion (p=0.029, Spearman’s rank correlation= -0.298) (**Figure 2A**), while no relation was found with total WBC (p=0.232, Spearman’s rank correlation= -0.154) (**Figure 2B**), BM blast proportion (p=0.315, Spearman’s Rho correlation: -0.138), FLT3 (p=0.284) and NPM1 (p=0.547) molecular status (**Supplementary Figure 2A**), cytogenetics (p=0.367), and 2017-ELN risk stratification groups (p=0.754) (**Supplementary Figures 2B, C**). As to survival outcomes, baseline vitamin C levels did not play a predictive role for overall survival (OS) nor disease-free survival (DFS) (**Supplementary Figure 3A, B**).

To explore the dynamics of vitamin C plasma levels with regards to disease status we longitudinally track its changes upon disease remission and relapse. To that end, we studied 13 AML patients at the time of achievement of CR as well as 10 patients with relapsed/refractory disease. As shown in **Figure 2C**, in patients who achieved CR, vitamin C levels significantly increased as compared to those at the time of diagnosis in serial patients (p=0.04), but overall were lower than those found in HDs (p=0.006). In contrast, we observed a further significant reduction of vitamin C concentration in the 10 relapsed/refractory patients when compared to corresponding samples at AML onset (p=0.002) (**Figure 2D**).

### Mutational Profile of AML Patients by Targeted-NGS

**Figure 3:**
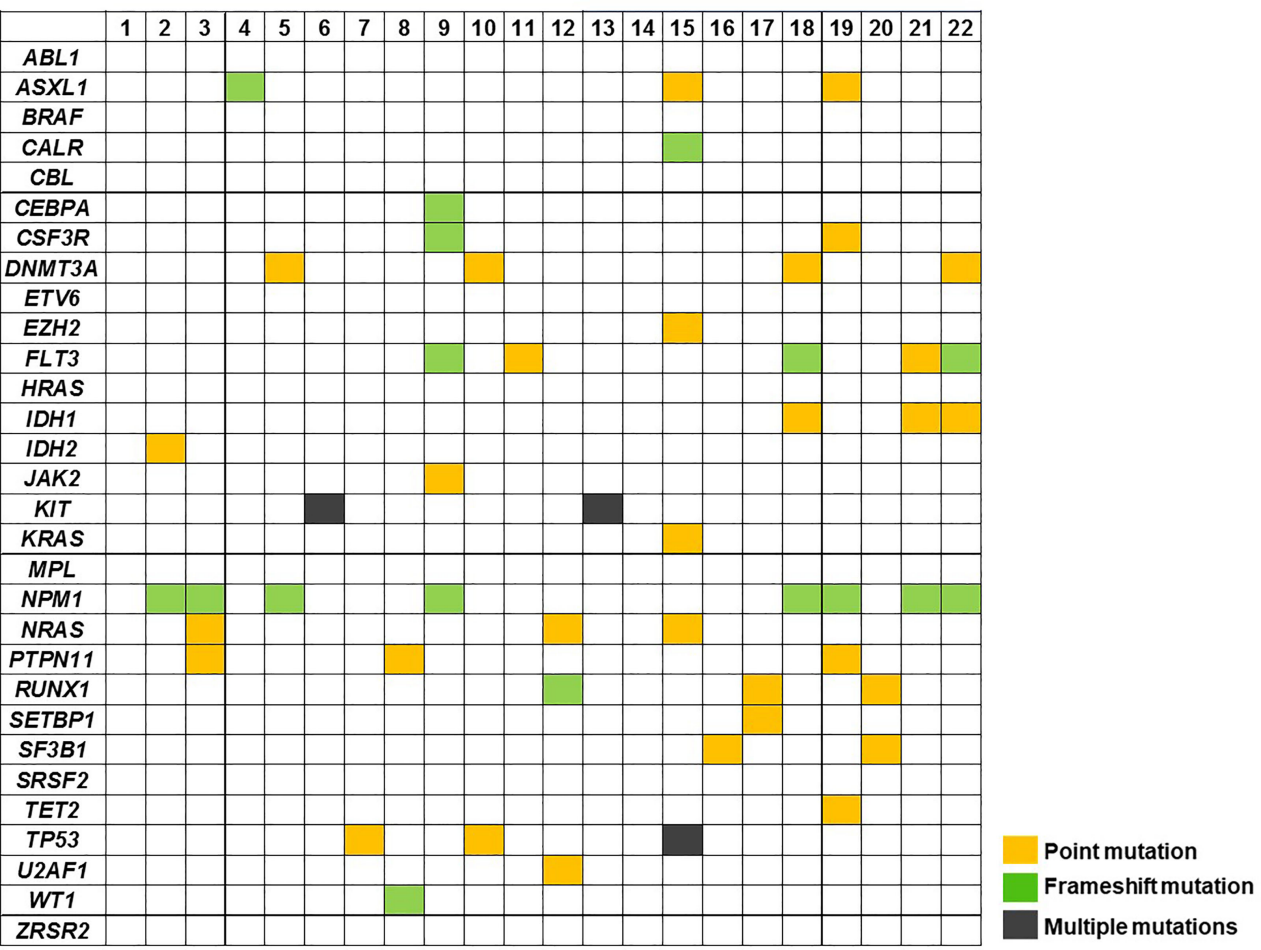
Mutation profile of AML cases at diagnosis (n= 22) using targeted NGS. Patient samples are displayed as columns.

Data on mutational status of common myeloid driver genes were available for 22 AML samples. A total of 54 mutations were found (median of 2.5 per patients) at a median VAF of 36.4% (range: 5.5-50.2%). Most common lesions were identified in NPM1 (n=8, 36%), FLT3 (n=5, 23%), DNMT3A (n=4, 19%) ASXL1, IDH1, NRAS, PTPN11, RUNX1 and TP53 (n=3 each, 14%) genes (**Figure 3**). No correlation between mutational profile and vitamin C concentration was found.

### Quantification of SCL23A2, SLC2A1, SLC2A3 Vitamin C Transporters, and TET2 Expression

**Figure 4:**
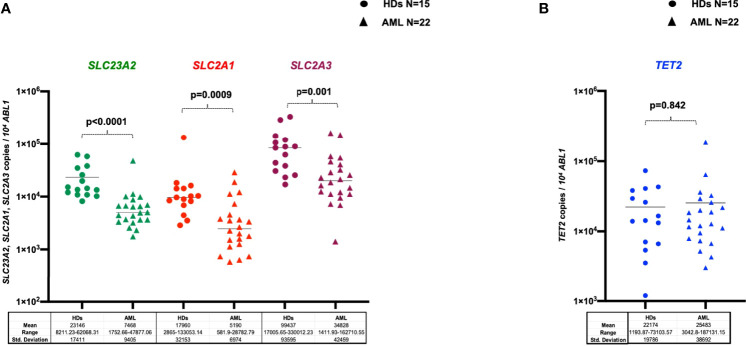
Levels of *SLC23A2*, *SLC2A1*, *SLC2A3* vitamin C transporters **(A)** and of *TET2* transcripts **(B)** evaluated by RQ-PCR in the PB-MNCs of healthy donors (n=15) or AML patients at diagnosis (n=22). HDs, healthy donors.

To explore whether the observed differences in vitamin C levels may support dysfunctions of physiological uptakes mechanisms, a quantitative assessment of SCL23A2, SLC2A1, and SLC2A3 transcripts was performed on PB-MNCs samples harvested at diagnosis from 22 AML cases and compared to those of 15 HDs. As reported in **Figure 4A**, expression of the main vitamin C transporters SCL23A2, SLC2A1, and SLC2A3 was significantly reduced in AML patients as compared to HDs (p<0.0001, p=0.0009 and p=0.001, respectively). Moreover, since it has been shown that vitamin C drives the expression of a TET2-dependent gene signature in human leukemia cell lines (25), we investigated, in the same cohort of AML patients and HDs, the TET2 transcripts level. We did not find any differences in TET2 expression between patients and controls (p=0.842) (**Figure 4B**). Moreover, no correlations between vitamin C concentration and SCL23A2, SLC2A1, SLC2A3 and TET2 levels in AML patients with available paired samples (data not shown) were found.

### Analysis of Intracellular Vitamin C Concentration and Uptake in AML Samples

**Figure 5:**
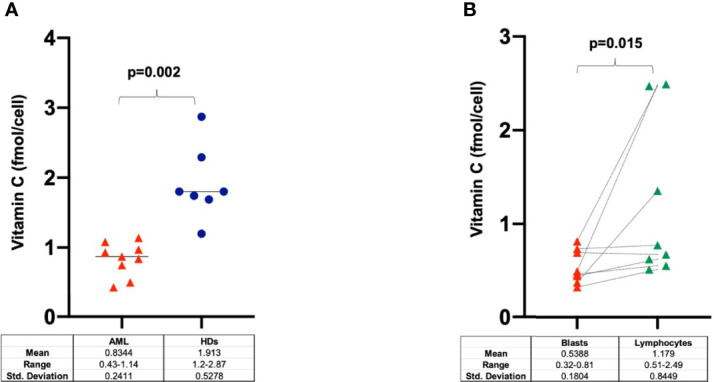
Intracellular concentration of vitamin C in **(A)** PB-MNCs at diagnosis (n=9), healthy donors (n=7) and in **(B)** blasts and lymphocytes purified from AML patients at disease onset (n=8). HDs, healthy donors.

Cytoplasmic vitamin C concentration was investigated in MNCs purified from AML samples at diagnosis (n=9), lymphocytes and blast cells (n=8) and HDs (n=7). As shown in **Figure 5A**, the intracellular concentration of vitamin C was significant lower in MNCs purified from AML patients as compared to HDs (p=0.002). To further identify the specific cellular fractions, AML blasts contained significantly less vitamin C than corresponding lymphocyte fractions (p=0.015) (**Figure 5B**).

**Figure 6:**
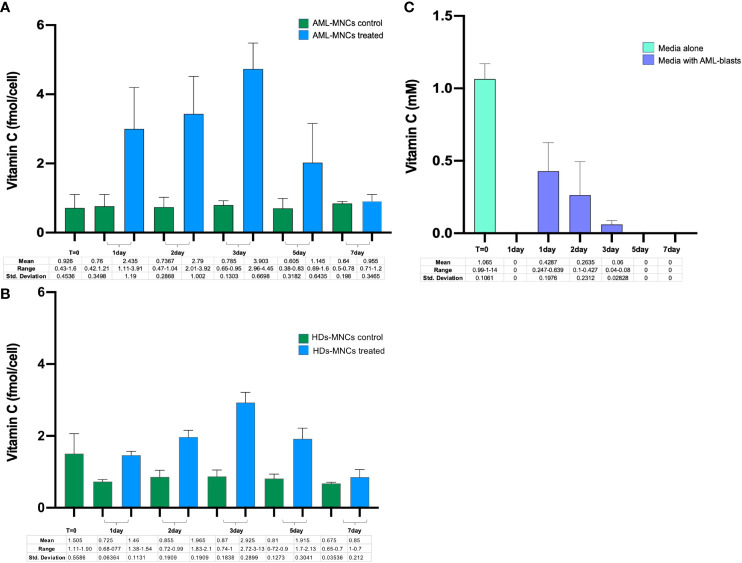
PB-MNCs were purified from AML samples at diagnosis and from healthy donors. In particular, 10^6^ primary cells/ml were cultured for one week with or without 1mM ascorbate and analyzed for vitamin C uptake at different time points. **(A)** Vitamin C uptake in untreated MNCs (green bar) and after incubation with 1mM ascorbate (blu bar) in AML (N=5) and **(B)** in HDs (N=2). **(C)** Concentration of vitamin C in culture medium with or without AML-MNCs. The light green bar (T=0) shows the level of vitamin C just after addiction of 1mM of ascorbate in the free cell culture medium. After one day in the incubator, vitamin C is anymore detectable. The purple bars show the vitamin C detected in culture medium after 1-7 days in presence of ascorbate treated MNCs. Data represent means of 5 *in vitro* experiments.

The flux of vitamin C through the cells is controlled by regulation of active and facilitated diffusion transport, mediated by SVCT and GLUT transporters, respectively. Since expression of the main vitamin C transporters and intracellular vitamin C concentration were low in AML patients, we investigated the ability of primary MNCs purified from 5 AML patients at onset, to absorb vitamin C by measuring its uptake across the cell membrane at 1, 2, 3, 5 and 7 days after incubation with 1mM ascorbate. As reported in **Figure 6A**, the vitamin C cellular uptake increased in a time-dependent manner, with maximum ascorbate incorporation after 72h. Vitamin C intracellular concentration progressively decreased, reaching the basal level value of untreated blasts after 5 and 7 days. The same experiment in PB-derived HD-MNCs showed a similar ascorbate uptake with a peak at 72h, albeit at a lower rate (**Figure 6B**). These results suggest that although primary AML blasts are characterized by low levels of SVCT and GLUT transporters, neoplastic cells are still able to absorb vitamin C and that vitamin C transporters are not the only factors dictating the ability of leukemic blasts to regulate such process. Moreover, vitamin C was undetectable in the culture medium just after 24h in the absence of cells, due to natural decay but, by contrast, was detectable in medium with cultured blasts until 72h indicating an active role of blasts to maintain vitamin C in the culture medium. Altogether, these findings underline a specific role of AML blasts in vitamin C turnover (**Figure 6C**).

## Discussion

Vitamin C has been shown to play a significant role as an epigenetic anticancer agent able to reprogram HSCs (20), suggesting that its administration may help to restore normal hematopoiesis (40). The role of vitamin C as an anti-cancer agent has long been debated (11, 22) and although recent reports have suggested it as a novel metabolic tumor suppressor, results on its clinical benefits remain controversial.

Despite current research, in the literature a scarcity of data exist on vitamin C status in patients with hematological malignancies (41–43), and to our knowledge there are no reports on the correlations with biological features and treatment response specifically in AML patients. A possible explanation is deriving from the technical difficulties of vitamin C sampling and processing, which require dedicated protocols and time-sensitive procedures.

We showed that vitamin C level were low in a large cohort of patients with a myeloid neoplasm at disease onset, including AML, APL and MDS. These data are in line with other reports on low vitamin C levels in patients with solid cancers (19). Furthermore, we demonstrated an inverse correlation between PB blast counts and vitamin C concentration in AML. The lower level of plasma vitamin C observed at diagnosis and in serial samples at disease progression may depend on its consumption by blasts. Indeed, vitamin C levels significantly increased in patients who achieved CR. This is in according with hypothesis proposed by Stone I (44). who suggested a role of the individual patients’ disease burden on further deepening the metabolic deficiency deriving by man’s inability to synthesize ascorbic acid. However, this concentration remained lower than that found in HDs samples, perhaps because of the intensity of the chemotherapy regimen received and the obvious nutritional impairment deriving from chemotherapy-related toxicities. Similar data has been reported in patients with advanced solid tumors (18, 45). In particular, Mayland C.R et al., reported vitamin C deficiency in 30% of patients with advanced cancers, including brain, bronchial, urogenital, and gastrointestinal neoplasms, admitted in a hospice setting, and found that patients with such a deficiency had a significantly shorter survival. In our patient cohort, we found no correlations between vitamin C levels and survival or cumulative incidence of relapse. However, differences in the characteristics of ours and the previous study cohort may explain such discrepancy. In fact, AML is an acute disorder which at diagnosis does not reflect the nutritional and metabolic changes possibly occurring in advanced, terminally ill patients with solid cancers.

Recently, the concept that vitamin C can be used to treat patients with cancer has been a hot topic for numerous lines of research (22, 46). Zhao et al., demonstrated that combination of vitamin C and decitabine activates TET2 in leukemia cells, improving OS in elderly patients. In particular, several authors showed that vitamin C may be a useful anti-leukemic agent able to restore the dioxygenase activity, thereby attenuating the effects of TET2 dysfunction, a widespread feature of hematological malignancies beyond the mere presence of mutations (25, 27, 29, 47–49). However, the degree of vitamin C absorption by leukemic cells is unknown. In the current study, we found that expression of SLC2A1, SCL2A3 and SCL23A2 transporters was lower in AML samples as compared to HDs. This result is in line with Liu J. et al. (50), who showed that SLC2A3 expression was significantly decreased in leukemic blasts, suggesting a defective ability to absorb vitamin C. In this line, cytoplasmic vitamin C levels were significant reduced in MNCs purified from AML patients as compared to lymphocytes and HDs. To validate this hypothesis, we studied vitamin C uptake by primary blasts in vitro. Our results, shower a significantly time-dependent intracellular increase of vitamin C, with maximum absorption after 72h, although vitamin C in the culture media has a very shorter half-life (<24h). These results may indicate either a higher metabolic turnover or an insufficient vitamin C compensation of TET2 dysfunction in AML blasts. Whatever the scenario is, metabolic pathways governing vitamin C uptake and its use are deregulated in AML, especially when considering physiological levels. To this end, the use of pharmacological vitamin C doses may be helpful in bypassing both the downregulation of specific transporters and the higher metabolic demand to restore TET2 dioxygenases functions. It is not a case that one of the anecdotal ascorbate-induced clinical remissions reported in the literature (51) was indeed associated with specific mutations in the IDH1/2-TET2-WT1 pathway and that current clinical trials are evaluating the role of ascorbate in specific population of patients (TET2-mutants) with myeloid disorders (ClinicalTrials.gov Identifier: NCT03397173), among others.

In conclusion, we add to the current literature on vitamin C by expanding our understanding of the longitudinal dynamics of this essential organic molecule in hematological malignancies, mainly AML. Our data show that despite the low levels of transporters, leukemic blasts are still able to adsorb and utilize vitamin C. However, probably due to the increased metabolic demand typical of rapidly proliferating cells, the intracellular levels remain low. Current research is trying to unravel the downstream effects of vitamin C and its role in blasts proliferation in a variety of scenarios. To that end, understanding the intertwining relationship between epigenetic changes, vitamin C and TET2 (with its pivotal role in myeloid malignancies) is crucial. Future studies exploring vitamin C metabolism in AML blasts are warranted to unravel its metabolic effects and role in blasts proliferation in a variety of scenarios (e.g., TET2-IDH1/2 mutants) to identify the categories of patients who may benefit the most from its therapeutic supplementation.

## Data Availability Statement

Targeted-NGS sequencing data are stored at https://www.sophiagenetics.com (SOPHiA DDM platform), and can be extracted using the Sophia-DDM-v4 password protected software. Raw data will be provided to Researchers upon request.

## Ethics Statement

The studies involving human participants were reviewed and approved by Comitato Etico Indipendente (D.M. 8 febbraio 2013); PROTOCOLLO DI STUDIO REGISTRO SPERIMENTAZIONI 154/17. Fondazione Policlinico Tor Vergata. The patients/participants provided their written informed consent to participate in this study.

## Author Contributions

TO, IF, LC, RM, AV, and MV made substantial contributions to conception, design of the study, analysis and interpretation of the data; GF, MD, ST, EB, MG, AN, FMo, and EF performed the experiments; FMa, RP, CG, RC, and EL performed the research and contributed patient samples and clinical data; MC provided experiments and critical revision of the manuscript; DA, MB, and LB performed cell sorting and FACS analyses. All authors contributed to the article and approved the submitted version.

## Funding

This work was supported by GR-2018-12365529-Santa Lucia to TO, AIRC 5×1000 call “Metastatic disease: the key unmet need in oncology” to MYNERVA project, #21267 (Myeloid Neoplasms Research Venture AIRC. A detailed description of the MYNERVA project is available at http://www.progettoagimm.it.) to MV, PRIN grant N. 2017WXR7ZT to MV and grant from the Ministero della Salute, Rome, Italy (Finalizzata 2018, NET‐2018‐12365935, Personalized medicine program on myeloid neoplasms: characterization of the patient’s genome for clinical decision making and systematic collection of real world data to improve quality of health care) to MV.

## Conflict of Interest

The authors declare that the research was conducted in the absence of any commercial or financial relationships that could be construed as a potential conflict of interest.

## Publisher’s Note

All claims expressed in this article are solely those of the authors and do not necessarily represent those of their affiliated organizations, or those of the publisher, the editors and the reviewers. Any product that may be evaluated in this article, or claim that may be made by its manufacturer, is not guaranteed or endorsed by the publisher.
