# Supplementary figures and images for: Vitamin C Deficiency in Patients With Acute Myeloid Leukemia
Source: Front Oncol. 2022 Jun 27;12:890344. doi: 10.3389/fonc.2022.890344 (PMC9271703; doi:10.3389/fonc.2022.890344)

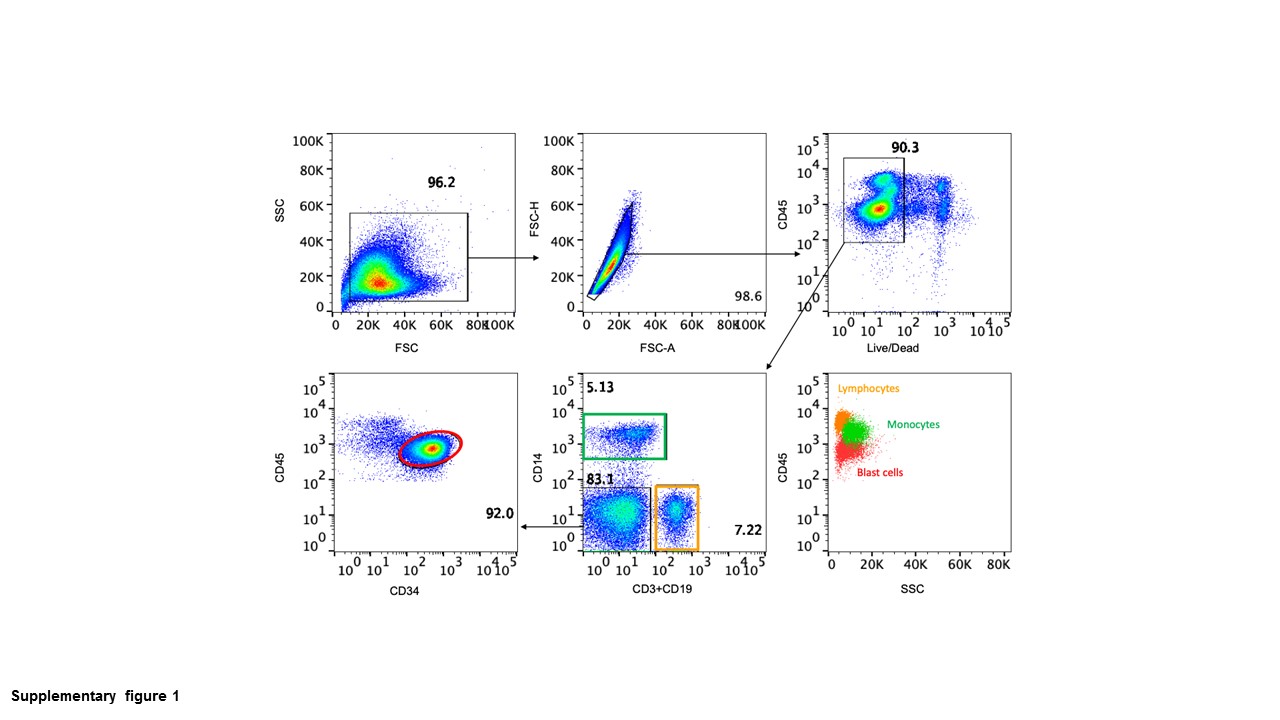

Supplement: Supplementary file 1 [file Image_1.jpeg]

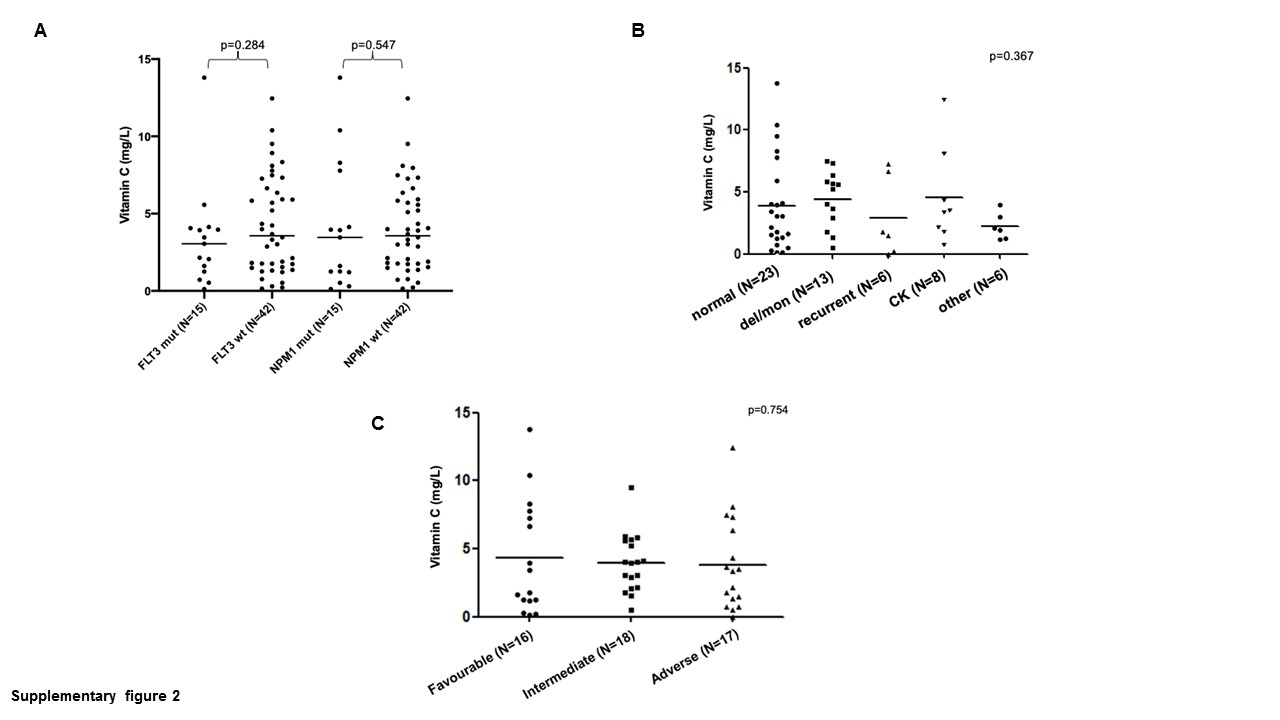

Supplement: Supplementary file 2 [file Image_2.jpeg]

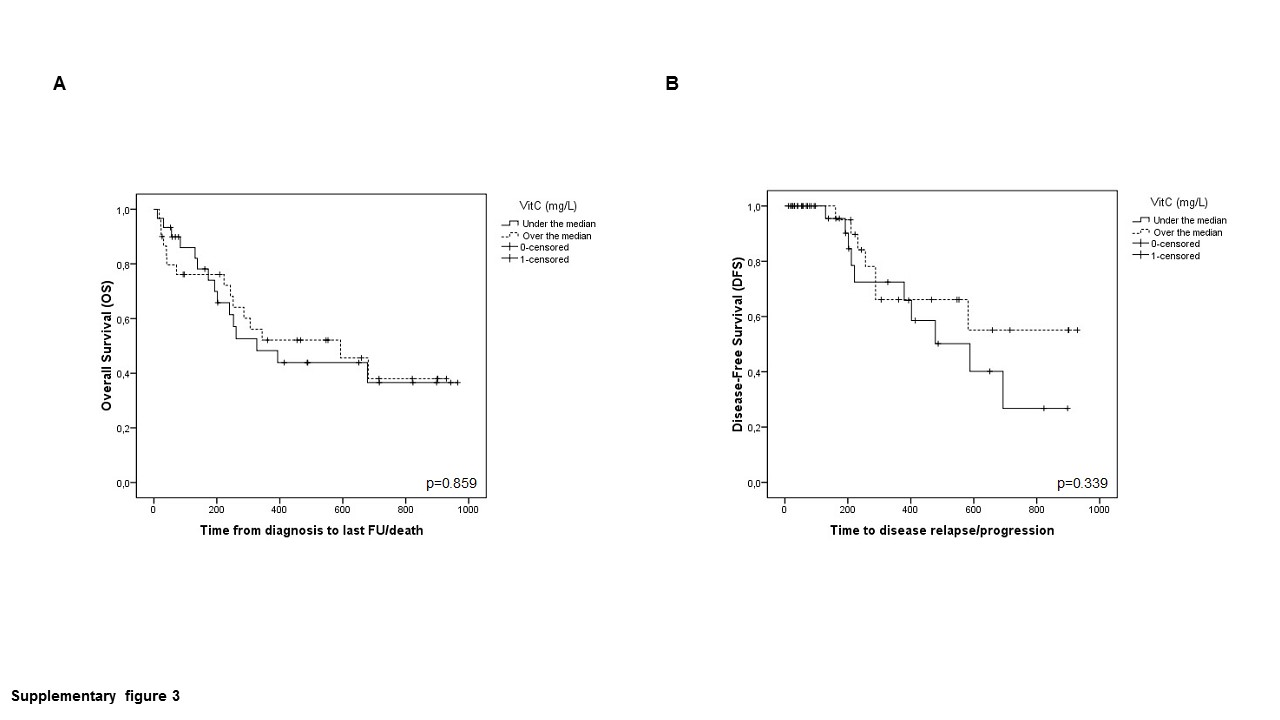

Supplement: Supplementary file 3 [file Image_3.jpeg]
